# Dopamine-Mediated Oxidation of Methionine 127 in α-Synuclein Causes Cytotoxicity and Oligomerization of α-Synuclein

**DOI:** 10.1371/journal.pone.0055068

**Published:** 2013-02-14

**Authors:** Kazuhiro Nakaso, Naoko Tajima, Satoru Ito, Mari Teraoka, Atsushi Yamashita, Yosuke Horikoshi, Daisuke Kikuchi, Shinsuke Mochida, Kenji Nakashima, Tatsuya Matsura

**Affiliations:** 1 Division of Medical Biochemistry, Department of Pathophysiological and Therapeutic Science, Tottori University Faculty of Medicine, Yonago, Japan; 2 Division of Neurology, Department of Brain and Neurosciences, Tottori University Faculty of Medicine, Yonago, Japan; 3 Division of Emergency and Disaster Medicine, Department of Surgery, Tottori University Faculty of Medicine, Yonago, Japan; 4 Division of Anesthesiology and Critical Medicine, Department of Surgery, Tottori University Faculty of Medicine, Yonago, Japan; University of Maryland School of Pharmacy, United States of America

## Abstract

Parkinson's disease (PD) is a neurodegenerative disorder characterized by the selective loss of dopaminergic neurons and the presence of Lewy bodies. Many recent studies focused on the interaction between α-synuclein (α-syn) and dopamine in the pathogenesis of PD, and fluorescent anisotropy suggested that the C-terminal region of α-syn may be a target for modification by dopamine. However, it is not well understood why PD-related pathogenesis occurs selectively in dopaminergic neurons. We investigated the interaction between dopamine and α-syn with regard to cytotoxicity. A soluble oligomer was formed by co-incubating α-syn and dopamine *in vitro*. To clarify the effect of dopamine on α-syn in cells, we generated PC12 cells expressing human α-syn, as well as the α-syn mutants, M116A, Y125D, M127A, S129A, and M116A/M127A, in a tetracycline-inducible manner (PC12-TetOFF-α-syn). Overexpression of wildtype α-syn in catecholaminergic PC12 cells decreased cell viability in long-term cultures, while a competitive inhibitor of tyrosine hydroxylase blocked this vulnerability, suggesting that α-syn-related cytotoxicity is associated with dopamine metabolism. The vulnerabilities of all mutant cell lines were lower than that of wildtype α-syn-expressing cells. Moreover, α-syn containing dopamine-mediated oxidized methionine (Met(O)) was detected in PC12-TetOFF-α-syn. Met(O) was lower in methionine mutant cells, especially in the M127A or M116A/M127A mutants, but also in the Y125D and S129A mutants. Co-incubation of dopamine and the _125_YEMPS_129_ peptide enhanced the production of H_2_O_2_, which may oxidize methionine residues and convert them to Met(O). Y125- or S129-lacking peptides did not enhance the dopamine-related production of H_2_O_2_. Our results suggest that M127 is the major target for oxidative modification by dopamine, and that Y125 and S129 may act as enhancers of this modification. These results may describe a mechanism of dopaminergic neuron-specific toxicity of α-syn in the pathogenesis of PD.

## Introduction

Parkinson's disease (PD) is one of the major neurodegenerative diseases, and is characterized by a selective degeneration of dopamine (DA) neurons and the formation of α-synuclein (α-syn)-containing Lewy bodies in the substantia nigra, with the subsequent loss of their terminals in the striatum [1], [2], [3]. The ensuing loss of DA causes most of the debilitating motor disturbances associated with PD. In addition to DA deficits, peripheral catecholaminergic neurons are also disturbed, resulting a severe autonomic disorder [4], [5], [6].

Numerous genetic risk factors are reported to induce and/or enhance PD onset [3], [7], [8], [9], [10], and α-syn is one of the most important molecules associated with the pathogenesis of familial and sporadic PD [9]. α-syn is a 140-amino acid protein that is a major component of Lewy bodies in the brains of PD patients as well as those with dementia with Lewy bodies [1], [11]. Pathogenic accumulation of α-syn occurs not only in the neuropil of the central nerve system, but also in peripheral catecholaminergic neurons [1], [10], [11], and in glial cytoplasmic inclusions in the brains of multiple system atrophy patients [12]. Together, these α-syn-related pathologies are known as “synucleinopathies.” α-syn is mainly localized in presynaptic terminals, and is a causative molecule for the PARK1 [13], [14] and PARK4 [15], [16], [17] forms of familial PD. It is reported to play an important cooperative role along with cysteine-string protein-α in protecting synapse function [18], and is also characterized as a protective molecule in non-DA/catecholaminergic cells. On the other hand, in DA cells α-syn acts as a toxic molecule [19]. For example in PARK4 patients, an overexpression of α-syn due to multiplication of the α-syn gene, induces cell loss of DA neurons and typical parkinsonism [15], [16], [17], and overexpression of α-syn with the pathogenic mutations found in PARK1 patients also results in DA-related cell death. We also reported that overexpression of α-syn in catecholaminergic PC12 cells leads to DA-related vulnerabilities against several toxicities including endoplasmic reticulum stress [20]. However, it is not well understood why DA neurons are specifically disturbed by the overexpression of α-syn. One possible mechanism is that oxidized forms of DA, such as dopamine quinone (DAQ), may interact with α-syn and induce its oligomerization [21].

The α-syn amino acid sequence is classified into three major regions: the N-terminal region (residues 1–60) contains a repetitive sequence including a KTKEGV motif; the middle part (residues 61–95) contains an NAC domain that forms the amyloidogenic core of α-syn; and the C-terminal sequence (residues 94–140) contains a negatively charged region [22]–[24]. Oligomerization of α-syn is believed to be an important step in its pathogenic toxicity, and several post-translational modifications, including phosphorylation [25], [26], nitration [27], and glycosylation [28], have been reported. However, these modifications are not specific to DA neurons. It was recently reported that oxidation of methionine residues in α-syn is required for oligomerization in the presence of DA *in vitro* [29], and that the C-terminal region of α-syn can loosely interact with dopachrome, thereby initiating oligomerization of α-syn [30]. On the other hand, Zhou et al. suggested that oxidation of α-syn methionines by H2O2 induced relatively stable α-syn oligomers, which are not toxic to DA neurons [31]. Thus, oxidation of α-syn methionine residues may be a key modification for DA neuron-specific vulnerabilities in the pathogenesis of PD. To investigate DA-related modifications of α-syn and the DA neuron-specific pathogenesis of PD, we established several cell lines expressing mutant forms of α-syn, and examined the role of Met(O) in α-syn oligomerization and cytotoxicity.

## Materials and Methods

### Chemicals and antibodies

DA and 3-(4,5-dimethylthiazol-2-yl) 2,5-diphenyl tetrasodium bromide (MTT) were obtained from Wako (Osaka, Japan). α-methyltyrosine (aMT) was purchased from Pfaltz &Bauer (Waterbury, CT, USA). Nerve growth factor (NGF) was purchased from Invitrogen (Carlsbad, CA, USA). Tet System Approved fetal bovine serum (FBS) and doxycycline (Dox) were obtained from Clontech (Palo Alto, CA, USA). Mouse monoclonal antibody against human α-syn and β-synuclein (β-syn) was purchased from BD Transduction Laboratories (Lexington, KY, USA). Rabbit polyclonal antibodies against methionine sulfoxide (Met(O)) and against β-actin were purchased from Novus Biologicals (Littleton, CO, USA) and Cell Signaling Technology (Beverly, MA, USA), respectively. HRP-linked anti-mouse IgG and anti-rabbit IgG were from Amersham Biosciences (Buckinghamshire, UK). Recombinant human α-syn was obtained from Bio Mol (Plymouth Meeting, MA). Designed peptides _125_YEMPS_129_, Fluorescein (Flu)-_125_YEMPS_129_, Flu-120PDNEA_124_, 122NEAYEMPSEEG_132_, _126_EMPSEEGY_133_, _122_NEAYEMP_128_, and mutant peptide _125_YEAPS_129_ (M127A) were purchased from Sigma Aldrich Japan (Tokyo, Japan).

### Biotinylation of α-syn and in vitro oligomerization analyses

Biotinylated α-syn was used for *in vitro* oligomerization analyses. Briefly, recombinant α-syn protein was biotinylated using the ECL Protein Biotinylation module (GE Healthcare UK Ltd, Buckinghamshire, England) according to the manufacturer's protocol. *In vitro* oligomerization was induced in a solution of 20 μM biotinylated α-syn, 10 mM Tris–HCl with several pH conditions, and 1 mM DA at 30°C for 12 h. The reaction mixture was lysed in an equal amount of 2×SDS sample buffer (100 mM Tris-HCl, pH 6.8, 4% SDS, 20% glycerol, 2 mM EDTA, 4 mM PMSF), and the soluble fraction was subjected to SDS-PAGE after centrifugation at 10,000×*g* for 5 min.

### SDS-PAGE and immunobloting

Samples from the *in vitro* oligomerization analyses were subjected to SDS-PAGE following standard procedures. After blotting onto PVDF membrane, biotinylated α-syn was detected by chemiluminescence using HRP-conjugated streptavidin (GE Healthcare) and ECL western blotting detection reagents (GE Healthcare).

For immunoblots from cell samples, cultured cells were harvested from 6-well culture plates, and lysed in SDS sample buffer (50 mM Tris-HCl, pH 6.8, 2% SDS, 10% glycerol, 1 mM PMSF, 2 mM EDTA). Aliguots (20 μg) were separated by molecular size on 12% polyacrylamide gels, transferred onto PVDF membrane (Hybond-P; GE Healthcare) and hybridized with the required antibody in PBS-Tween 20 at room temperature for 1 h. The immunoreactive signal was detected using HRP-linked anti-rabbit IgG and ECL detection reagents (GE Healthcare).

### Fluorescence anisotropy assays

For semiquantitative analyses of binding between the α-syn sequence _125_YEMPS_129_ and DA or its metabolites, a fluorescence anisotropy-based assay was employed. Fluorescein (Flu)-labeled _125_YEMPS_129_ peptide, or _120_PDNEA_124_ peptide as a control (CTL), was incubated at room temperature with or without DA under various pH conditions. Fluorescence anisotropy was measured using fluorescein excitation (λ_ex_ = 490 nm) and emission (λ_em_ = 535 nm) filters and a Beacon 2000 Fluorescence Polarization system (Hybrid Instruments Japan Co., Osaka, Japan).

### Establishment of α-synuclein- or β-synuclein-expressing cell lines and cell cultures

PC12 cells expressing human α-syn or β-syn controlled by the Tet OFF system (PC12-TetOFF-α-syn, PC12-TetOFF-β-syn) were established following the procedure described previously [21]. Briefly, cDNA of human α-syn was amplified from cDNAs raised from human brain tissue using the primer set 5′-ATGGATGTATTCATGAAAGGACTTTCA-3′ and 5′-TTAGGCTTCAGGTTCGTACTCTTG-3′ by RT-PCR. For the β-syn-expressing cell line, cDNA of β-syn was subcloned from human brain cDNAs by RT-PCR using 5′-AAGCTTAGGATGGACGTGTTC-3′ and 5′-ACTACGCCTCTGGCTCATA-3′. The PCR products were subcloned into the pGEM easy vector (Promega Corporation, Madison, WI, USA), and sequences were confirmed. The cDNAs of human α-syn or β-syn were then transferred into a pTRE2 vector (Clontech Laboratories Inc, Palo Alto, CA, USA). Mutageneses for M116A, Y125D, M127A, S129A, and M116A/M127A mutant cell lines were carried out using the pTRE2-wildtype α-syn construct as a template and the Site Directed Mutagenesis Kit (Stratagene, La Jolla, CA, USA) following the manufacturer's protocol. The primer sets for mutageneses are listed in Table 1. The M116A/M127A double mutant α-syn construct was prepared using the M127A mutant construct as a template. During the amplification of PC12-α-syn-Tet Off cells, they were maintained at 37°C in 5% CO_2_ in DMEM/F12 medium, supplemented with 5% FBS, 10,000 unit/ml penicillin G (PG), 100 mg/ml streptomycin (SM), and 2 ng/ml Dox. To induce α-syn expression, the culture medium was changed to Dox-free medium (DMEM/F12, 2.5% Tet System Approved FBS, and PG/SM). To induce neural differentiation, 50 ng/ml NGF was added during culturing. The culture medium was exchanged for fresh medium to completely delete Dox. For the present experiments, the cells were cultured for 1–8 days under Dox-free conditions.

**Table 1:** The primer sets used in the site directed mutageneses.

	primer sequence
Y125D Fw	5′-AATGAGGCTGATGAAATGCCTTCT-3'
Y125D Rv	5′-AGGCATTTCATCAGCCTCATTGTC-3'
S129A Fw	5′-GAAATGCCTGCTGAGGAAGGGTAT-3'
S129A Rv	5′-CCCTTCCTCAGCAGGCATTTCAT-3'
M116A Fw	5′-ATTCTGGAAGATGCGCCTGTGGATCCTGA-3'
M116A Rv	5′-AGGATCCACAGGCGCATCTTCCAGAATTCCTT-3'
M127A Fw	5′-AATGAGGCTTATGAAGCGCCTTCTGAGGAAGGG-3′
M127A Rv	5′-CCCTTCCTCAGAAGGCGCTTCATAAGCCTCATT-3'

### Cell viability assay (MTT assay)

Cell viability was measured using the MTT assay following the protocol described previously with some modifications [21]. For the MTT assay, 1×10^3^ cells/well were seeded in 96-well plates, cultured for 4 or 8 days, and then incubated with MTT for 2 h at 37°C. After adding 100 μl of 0.05 N HCl in 2-propanol and mixing thoroughly to dissolve the dark blue crystal, the MTT reduction was measured with a microplate reader (Bio-Rad; wavelength of 570 nm). The data are presented as percent post-treatment recovery (percent live cells), where the absorbance from the control, non-treated cells was defined as 100% live cells. For analyzing the effects of catecholamines on α-syn cytotoxicity in PC12 cells, PC12 cells were cultured with or without aMT, a specific inhibitor of tyrosine hydroxylase.

### Immunoprecipitation

Cells for immunoprecipitation were harvested and lysed in IP-lysis buffer (20 mM HEPES, pH 7.5, 150 mM NaCl, 1 mM EDTA, 10 μg/ml leupeptin, 1 mM PMSF, 1% Triton-X100, 0.1% SDS). The lysates were centrifuged at 12,000×*g* for 10 min, and the supernatant was transferred to another tube containing prepared Agarose-Protein G (Santa Cruz)-antibody conjugate. The mixture was incubated at 4°C for 12 h with gentle rotation, centrifuged at 5,000×*g* for 30 s, and the supernatant was discarded. The Protein A/G-agarose was washed 5 times with IP-lysis buffer and, finally, with IP-final washing buffer (20 mM HEPES, pH 7.5, 150 mM NaCl). The washed preparation was analyzed by western blotting using SDS sample buffer.

### Measurement of hydrogen peroxide *in vitro*

*In vitro* hydrogen peroxide (H_2_O_2_) levels were measured using the Amplite^TM^ Fluorimetric Hydrogen Peroxide Assay Kit (AAT Bioquest, Inc., Sunnyvale, CA, USA) following the manufacturer's protocol with some modifications. Briefly, 200 μM DA was incubated in 10 mM Tris-HCl, pH 7.4 with or without C-terminal region peptides of human α-syn, 0.4 U HRP, and 0.5× Amplite^TM^ Red peroxidase substrate stock solution in the kit. Reaction mixtures (100 μl each in 96-well plate) were incubated at 37°C for 30 min, and absorbance was measured at 562 nm using an absorbance microplate reader.

## Results

### DA enhances the oligomerization of α-syn *in vitro*

**Figure 1:**
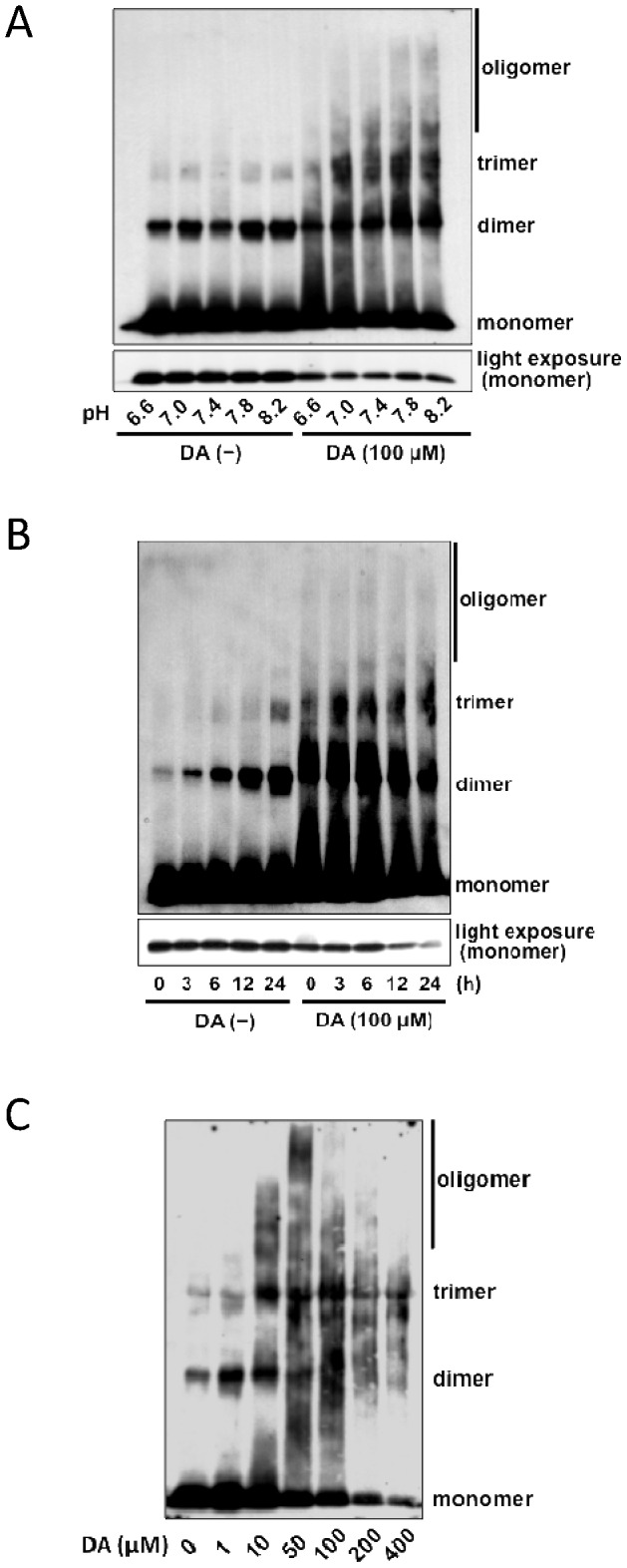
DA enhances oligomerization of α-syn *in vitro*. (A) Western blots showing that the oligomerization of biotinylated α-syn is enhanced by co-incubation with DA, especially under alkaline conditions (24 h). Biotinylated α-syn (20 μM) was incubated with (100 µM) and without (−) DA under several pH conditions (6.6–8.2). α-syn incubated with DA resulted in greater oligomerization than the α-syn in the absence of DA. The bottom panel shows the monomer α-syn visualized by light exposure when the film was developed. (B) Time course of oligomerization of α-syn. Although α-syn formed some oligomers gradually in the absence of DA (−), α-syn oligomerized much more rapidly in the presence of DA (100 μM, pH 8.2). The bottom panel shows the monomer α-syn visualized by light exposure when the film was developed, which decreased as the oligomer increased. (C) The oligomerization or aggregation of α-syn was DA-dose dependent (pH 8.2, 24 h). Excess concentrations of DA resulted in insoluble aggregates and reduced the amounts of soluble oligomer and monomer.

In our previous study, we showed that DA and DOPA enhanced the oligomerization of α-syn under alkaline conditions *in vitro* [21]. To further clarify the mechanisms by which DA enhances α-syn oligomerization, we observed the oligomerization of biotinylated α-syn under several different pH conditions, several durations, and several doses of DA (Fig. 1). Even in the absence of DA, α-syn oligomerized *in vitro*; however, the presence of DA enhanced the oligomerization of α-syn, especially under alkaline pH (Fig. 1A). In the absence of DA, oligomerization of α-syn was observed as soon as 3 h in pH 8.2 buffer, and the amount of soluble oligomers increased gradually. The presence of DA resulted in much earlier formation of soluble oligomers, and at the same time, decreased amounts of α-syn monomer (Fig. 1B). The oligomerization of α-syn was also dependent on the dose of DA (Fig. 1C), with higher concentrations of DA being more effective at inducing α-syn oligomerization; however, excess DA (200-400 μM) induced the formation of insoluble aggregates of α-syn, and both soluble oligomers and monomers decreased (Fig. 1C).

### Amino acid sequence _125_YEMPS_129_ of α-syn is important for the interaction between DA and α-syn

**Figure 2:**
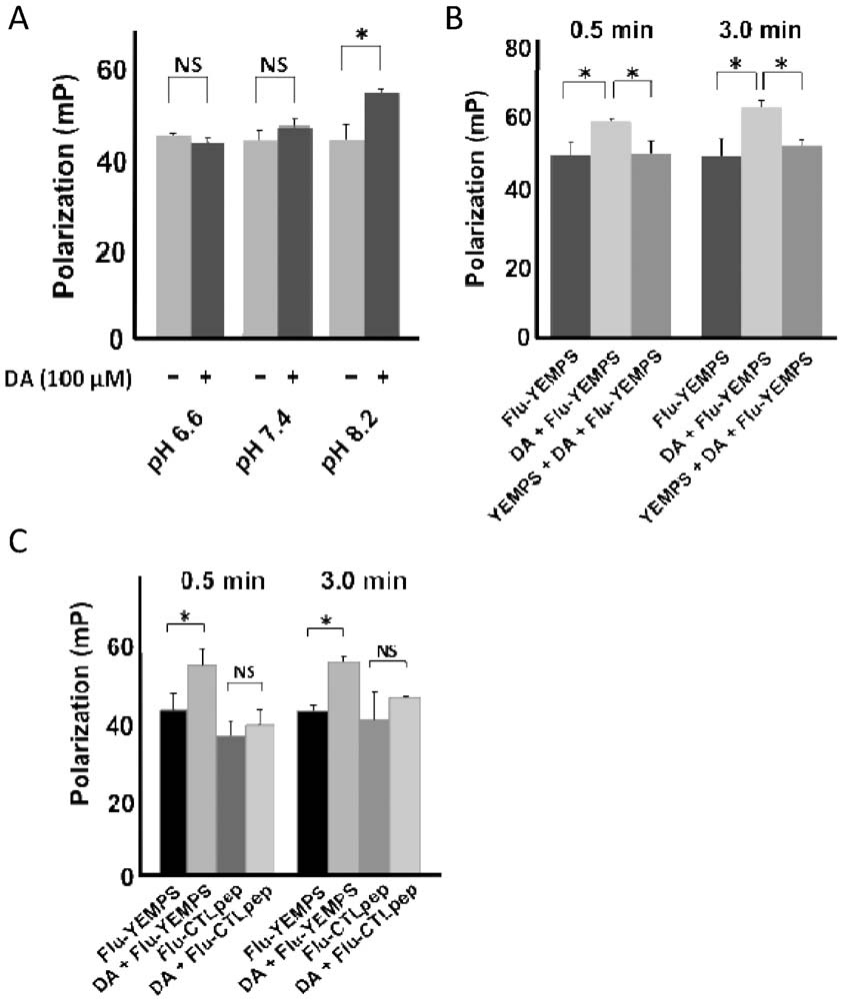
Interaction of DA with 125YEMPS129 measured by alterations in fluorescent anisotropy. (A) The anisotropic signal of the Flu-YEMPS peptide was altered by co-incubation with DA (100 μM, 3 min) at alkaline pH. *p<0.01 (ANOVA), NS: significant. (B) Non-labeled YEMPS competitively inhibited the DA-induced (100 μM, pH 8.2) alteration of the anisotropic signal of Flu-YEMPS *p<0.01. (C) The anisotropic signal of the Flu-120PDNEA124 control peptide (CTLpep) was not altered by the presence of DA (100 μM, pH 8.2) *p<0.01, NS: not significant.

A previous report suggested that the α-syn amino acid sequence including _125_YEMPS_129_ may play important roles in the DA- or its oxidative metabolites-mediated cytotoxicity of α-syn [30]. To clarify whether _125_YEMPS_129_ interacts with DA or its oxidative metabolites, we measured changes in fluorescent anisotropy signals of Flu-_125_YEMPS_129_ or the control peptide, Flu-_120_PDNEA_124_ in the presence or absence of DA. First, we investigated the effect of pH on the interaction between _125_YEMPS_129_ and DA. Changes in polarization were observed under alkaline conditions (pH 8.2) that can enhance the conversion of DA to its oxidative metabolites (Fig. 2A). The polarization change occurred in the first few minutes, and most of the alteration was completed 0.5 min into the co-incubation of Flu-_125_YEMPS_129_ and DA under pH 8.2 (Fig. 2B). Furthermore, non-labeled _125_YEMPS_129_ competitively inhibited the polarization change (Fig. 2B). We also investigated whether another α-syn amino acid sequence interacts with DA using Flu-_120_PDNEA_124_ as a control peptide; however, no significant polarization changes were observed (Fig 2C).

### DA-related cytotoxic mechanisms of α-syn in PC12 cells

**Figure 3:**
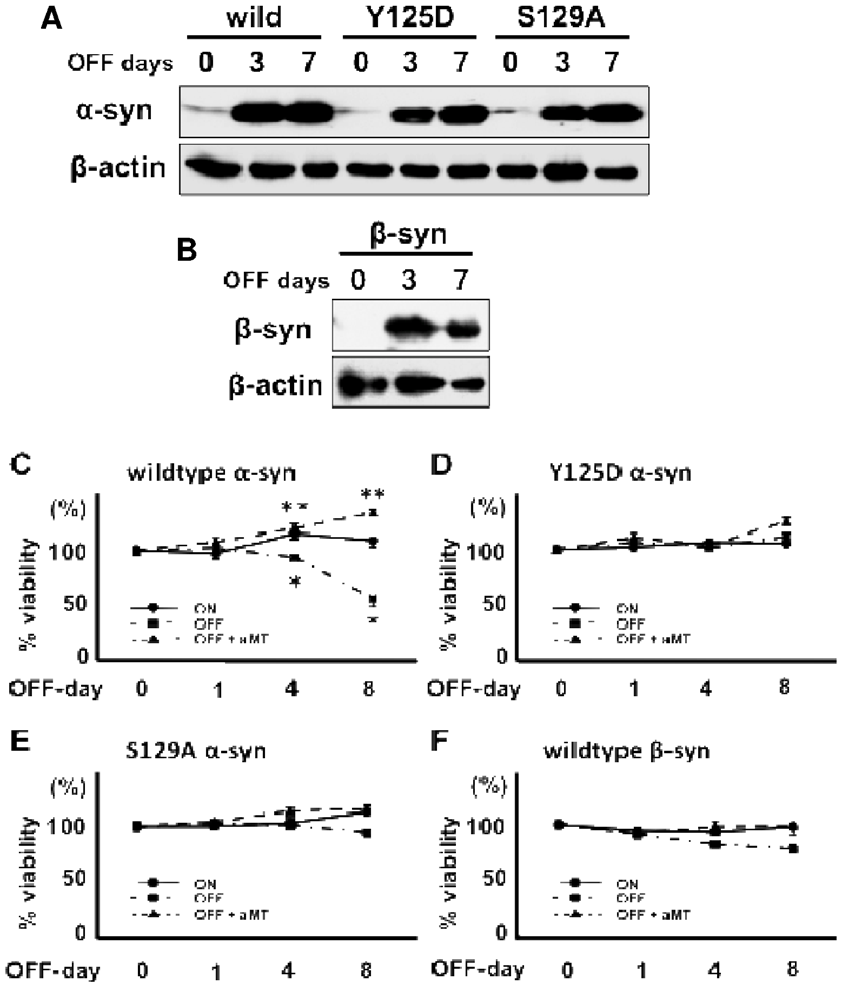
Catecholamine-associated cytotoxicity of α-syn in PC12 cells. **** (A) Western blotting for α-syn expression in Tet-Off-α-syn cells. Withdrawal of Dox (OFF days) from the medium induced the expression of α-syn at day 3, which increased by day 7 after Dox withdrawal. β-actin expression was used as an internal control. (B) Western blotting for β-syn expression in Tet-Off-β-syn cells. Withdrawal of Dox from the medium induced the expression of β-syn 3 and 7 days after Dox withdrawal. β-actin expression was used as an internal control. (C-F) Cell viability measured using the MTT assay (C: wildtype α-syn, D: Y125D-mutant α-syn, E: S129A-mutant α-syn, F: wildtype β-syn). (C) The expression of α-syn following withdrawal of Dox (OFF) significantly reduced cell viability compared with the α-syn-suppressed condition (ON). The inhibition of catecholamine metabolism by aMT (100 μM) dramatically raised cell viability even when α-syn expression was induced. *p<0.01 vs. ON, **p<0.01 vs. OFF (ANOVA). (D–F) The induction of Y125D α-syn- (D), S129A α-syn- (E) and β-syn- (F) expression by withdrawal of Dox (OFF) did not significantly alter cell viability compared to the respective α- or β-syn-suppressed conditions (ON). The inhibition of catecholamine metabolism by aMT (100 μM) also had no effect.

To examine the DA-related cytotoxicity of α-syn, we established α-syn-expressing PC12 cells using the Tet-OFF system. We also established Y125D- and S129A- mutant α-syn expressing PC12 cells as well as β-syn-expressing PC12 cells using the Tet-OFF system. Expressions of all three α-syn and the β-syn proteins were clearly induced 3 and 7 days following the withdrawal of Dox from the medium (Fig. 3A,B). In the wildtype α-syn-expressing cells, cell viability was decreased 4 or 8 days after the induction of α-syn expression (Fig. 3C). On the other hand, the inhibition of catecholamine metabolism by aMT blocked the cytotoxicity resulting from induced α-syn expression (Fig. 3C). In Y125D- and S129A-mutant α-syn-expressing and or β-syn-expressing cells, cell viability was not significantly decreased by DA-related cytotoxicity of α-syn, even when α-syn or β-syn were dramatically induced (Fig. 3D,E,F).

Because phosphorylations of the serine residue at position 129 (S129) and the tyrosine residue at the position 125 (Y125) were identified as pathogenic modifications in the pathogenesis of synucleinopathy, we examined the phosphorylation of S129 and Y125 using phospho-S129- or phospho-Y125- specific antibodies. However, no significant phosphorylation of either S129 or Y125 were observed in the presence of DA, or with aMT-treatment (data not shown).

### The Methionine residue at position 127 is important for DA-related cytotoxicity of α-syn in PC12 cells

**Figure 4:**
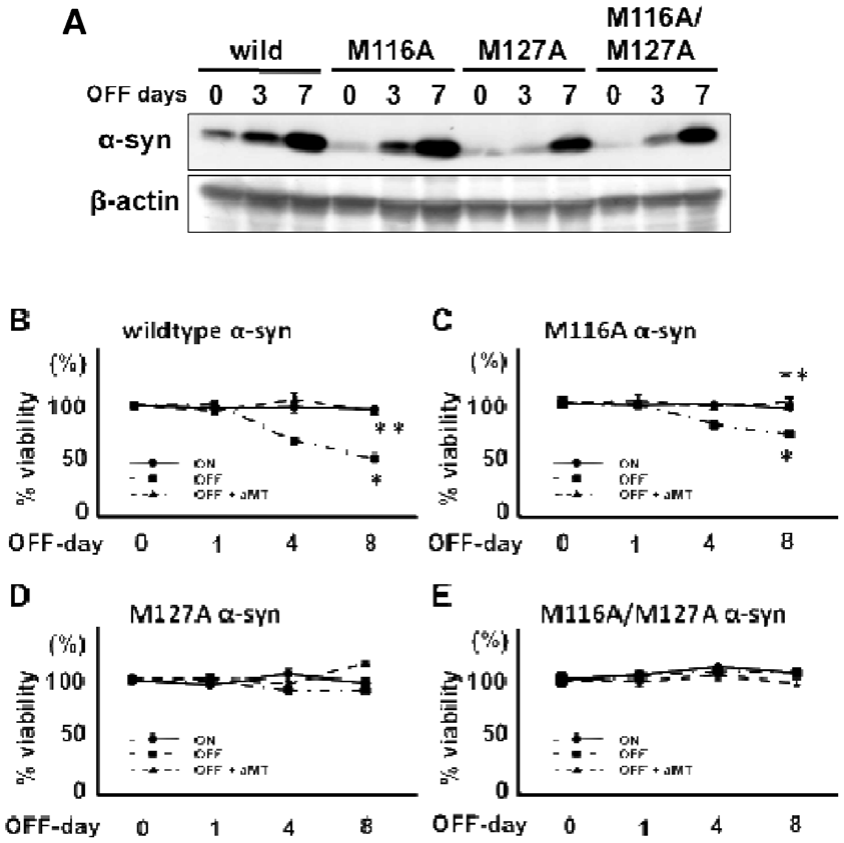
Catecholamine-associated cytotoxicity of α-syn and methionine mutant α-syn in PC12 cells. (A) Western blotting of wildtype (wild), and mutant (M116A, M127A, and M116A/M127A) α-syn expression in Tet-Off-α-syn cells. Withdrawal of Dox from the medium induced α-syn expression 3–7 days after Dox withdrawal. β-actin expression was used as an internal control. (B–E) Cell viability using the MTT assay (B: wildtype α-syn, C: M116A α-syn, D: M127A α-syn, E: M116A/M127A α-syn). (B) The expression of wildtype α-syn following withdrawal of Dox (OFF) reduced cell viability compared to the α-syn-suppressed condition (ON), while inhibition of catecholamine metabolism by aMT (100μM) dramatically blocked this cytotoxicity. *p<0.01 vs. ON, **p<0.01 vs. OFF. (C) The expression of M116A α-syn following withdrawal of Dox (OFF) also reduced cell viability compared to the α-syn-suppressed condition (ON), but to a lesser degree than wildtype α-syn, and aMT (100 μM) blocked this cytotoxicity. *p<0.01 vs. ON, **p<0.01 vs. OFF). (D,E) By contrast, expression of the M127A mutant (D) or the combined M116A/M127A (E) mutant α-syn did not reduce cell viability compared to the respective α-syn-suppressed condition (ON), and aMT (100 μM) also had no effect on either.

It was reported that methionine residues in α-syn are oxidized in the presence of DA; thus, we examined the effects of mutating methionine residues 116 (M116) and 127 (M127) on α-syn-cytotoxicity in M116A, M127A, and M116A/M127A mutant α-syn-expressing PC12 cells. All three α-syn mutants were induced 3–7 days after the withdrawal of Dox from the medium (Fig. 4A). Induced expression of M116A mutant α-syn was less toxic than the wildtype α-syn (compare Fig. 4B and C), while the M127A and M116A/M127A mutant cell lines showed no vulnerability to increased expression of respective α-syn mutants.

### Methionine sulfoxide is detected in α-syn in PC12 cells

**Figure 5:**
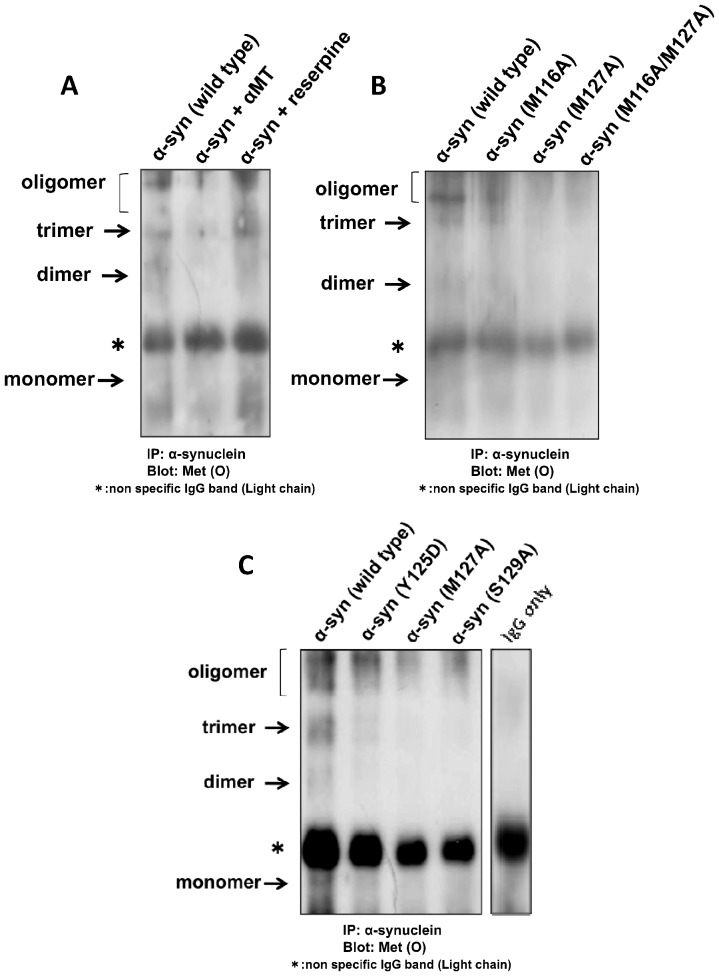
Detection of Met(O) in α-syn. (A) Western blots of α-syn immunoprecipitated (IP) from wildtype α-syn-expressing PC12 cells and immunostained with antibodies against Met(O). Met(O)-labeling was detected in the α-syn from PC12 cells with normal CA metabolism (left lane), but not in the α-syn from those treated with the tyrosine hydroxylase inhibitor aMT (100 μM) (middle lane). By contrast, Met(O) levels were enhanced by treatment with reserpine, an inhibitor of monoamine transport from cytosol to synaptic vesicles (right lane). (B) Met(O)-labeling in lines of PC12 cells expressing methionine mutants of α-syn. Lower levels of Met(O) were detected in the α-syn proteins with methionine mutations (M116A, M127A, M116A/M127A), especially in the M127A or M116A/M127A mutants of α-syn, even though catecholamine metabolism was not suppressed. (C) Met(O)-labeling in lines of PC12 cells expressing various mutants of α-syn. Lower levels of Met(O) were detected in the α-syn proteins with tyrosine (Y125D) and serine (S129A) mutations, compared with wildtype α-syn even though catecholamine metabolism was not suppressed. The lane for IgG control is also exhibited in order to prove the oligomer band is specific for α-syn.

In the presence of DA, Met(O) was reported to be an important modification of α-syn [30]; thus, we used anti-Met(O) antibodies to investigate whether α-syn Met(O) was present in our cell lines. Following immunoprecipitation of total α-syn from PC12 cells, we observed Met(O)-labeled α-syn that was decreased by the inhibition of catecholamine metabolism with aMT. On the other hand, reserpine, which increases intracellular free DA, increased the amount of Met(O)-labeled α-syn (Fig. 5A).

To identify which α-syn methionine residue is predominantly oxidized in the presence of DA, we compared the degree of α-syn methionine oxidation in wildtype α-syn-, M116A-, M127A-, and M116A/M127A-expressing PC12 cells. Each of the α-syn mutants, but particularly those with M127A mutations, exhibited less Met(O) than that in the wildtype α-syn, suggesting that M127 was the major target for methionine oxidation in α-syn.

Despite the undetectable levels of phosphorylation of the wildtype α-syn Y125 and S129 residues, mutating the tyrosine (Y125D) and serine (S129A) residues in the mutant α-syn-expressing PC12 cells still reduced DA-associated cell vulnerability (Fig. 3). In an attempt to explain this, we also checked for the presence of Met(O) in the Y125D and S129A mutants of α-syn. Surprisingly, Met(O) levels were lower in both the Y125D and S129A mutants of α-syn compared with wildtype, suggesting that the Y125 and/or S129 may also play an important role in the oxidation of M127 by DA (Fig. 5C).

### _125_YEMPS_129_ and _125_YEAPS_129_ (M127A) peptides enhance DAQ-related H_2_O_2_ formation in vitro

**Figure 6:**
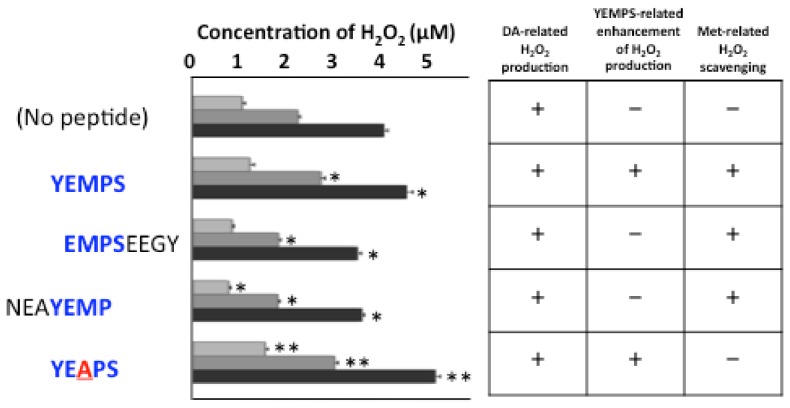
Concentrations of H_2_O_2_ produced by DA alone and with various peptides from the C-terminal sequence of α-syn 15 min (light gray), 30 min (dark gray), and 60 min (black) after incubation. DA alone (no peptide) produced increasing amounts of H_2_O_2_ over time. Co-incubation of DA with the YEMPS peptide enhanced H_2_O_2_ production, while the Y127- (EMPSEEGY) and S129- (NEAYEMP) lacking peptides produced less H_2_O_2_ than DA alone or with the YEMPS peptide. Moreover, co-incubation of DA with the methionine-mutated peptide (YEAPS) produced the highest amounts of H_2_O_2_ among all conditions. *p<0.01 vs. no peptide, **p<0.01 vs. no peptide and YEMPS (ANOVA).

Because the sulfide moiety in the methionine residue is usually oxidized by H_2_O_2_ when Met(O) is formed, we measured the levels of H_2_O_2_ production during the incubation of DA alone or with peptides designed from the amino acid sequence in the C-terminal of α-syn (Fig. 6). H_2_O_2_ was produced *in vitro* during the incubation of the reaction mixture (see Methods) with DA alone, and the co-incubation of DA with the _125_YEMPS_129_ peptide increased the production of H_2_O_2_, while the peptides lacking the Y125 or S129 residues decreased the production of H_2_O_2_ in the present experiments. Interestingly, co-incubation of DA with the mutant peptide _125_YEAPS_129_ (M127A) produced the highest levels of H_2_O_2_.

## Discussion

α-syn is a key molecule in PD pathogenesis, and is associates not only with familial PD, but also with Lewy body formation in the brains of sporadic PD patients [1], [2], [3]. Overexpression of α-syn in the PARK4 family of PD results in a typical parkinsonian phenotype and loss of dopaminergic neurons [15], [16], [17] as well as common sporadic PD. Some reports suggest that α-syn exhibits its cytotoxic character only in the dopaminergic cells, and not in the non-dopaminergic cells [19]. However, it is not well understood why high levels of α-syn expression induce selective cell death of the dopaminergic neurons. Conway reported that some compounds with catechol structures, including DA and DOPA, form adducts with α-syn, and that its unstable protofibril may be one of the causes of α-syn-related toxicity [32]. Indeed, one report suggests that DA and/or catecholamine-associated molecules interact with α-syn, especially with its C-terminal sequence [30]. Some studies using α-syn-null mutant mice also suggest that α-syn plays important roles in DA metabolism and has functional interactions with DA [33], [34].

We previously reported that the oxidized form of DA, DAQ, can interact directly with α-syn, inducing the oligomerization of α-syn *in vitro* [21]. Moreover, associations between DA/DAQ and various PD-related molecules, including parkin [35], tyrosine hydroxylase [36], the dopamine transporter [37], α-syn [38], [39], and ubiquitin [40], have been reported. These results suggest that the functional interaction between DA (and/or related molecules) and α-syn should be considered important in the pathogenesis in PD.

We previously established PC12-α-syn-Tet Off cells, in which expression of α-syn can be controlled by the withdrawal of Dox from the culture medium [20], and catecholamine metabolism can also be inhibited by aMT. Therefore, it may be a useful model for investigating pathogenic interactions between DA and α-syn. Using this cell line, we confirmed a catecholamine metabolism-related cytotoxicity of α-syn.

Post-translational modification of α-syn is an important step in the pathogenesis of PD [41]. Recently, oxidative modification of α-syn was the focus of possible DA-associated modification of α-syn [29], [42]. Methionine residues in proteins are oxidized by reactive oxygen species such as H_2_O_2_, and are converted to Met(O) [43]. The levels of Met(O) in several cellular proteins are also controlled by methionine sulfoxide reductase A (MsrA) and B (MsrB), and MsrA deficiency may enhance the stability of α-syn [44] and α-syn-related cytotoxicity [45], [46].

In the present study, we demonstrated that DA/DOPA must be oxidized for α-syn to form oligomers. Indeed, we recently showed that the oxidization of DA and DOPA are responsible for oligomerization of α-syn [21]. DA and DOPA are known to be oxidized to DAQ and DOPAQ, which are subsequently auto-oxidized to DA-chrome and melanin, respectively [47]. Such oxidized forms of DA/DOPA are believed to be toxic for neuronal cells; however, detailed mechanisms of how the combination of DA/DOPA and α-syn induce cellular toxicity are lacking. We investigated the association between DA/DOPA oxidation and α-syn oligomerization in a cell-free *in vitro* system and in Tet-controllable α-syn-expressing cells. The experiments showed that the co-existence of DA and α-syn resulted in the formation of soluble oligomers of α-syn, especially under alkaline conditions, suggesting that oxidized DA may enhance the oligomerization of α-syn. As some reports suggested that the C-terminal region of α-syn is important for the DA-related toxicity of α-syn [30], [32], we used fluorescent anisotropy to investigate which part of the α-syn molecule was targeted by oxidized DA. The results of these experiments suggest that the _125_YEMPS_129_ sequence close to the C-terminal of α-syn is a candidate target for oxidized DA. We also showed a direct interaction of oxidized DA with α-syn, which may be an initial step in α-syn oligomerization in the presence of DA [21]. Because we believe that α-syn must be toxic in dopaminergic and/or catecholaminergic cells, it may be difficult to maintain the expression of α-syn in such cells; therefore, we established PC12 cell lines, in which the expression level of α-syn was controlled by the withdrawal of Dox using the Tet-Off system [20], [29]. We also prepared mutant α-syn-expressing cells (Y125D, S129A, M116A, M127A, M116A/M127A) as well as β-syn-expressing cells. α-syn was cytotoxic in the presence of the normally existing catecholamines in PC12 cells, but not when catecholamine metabolism was inhibited with aMT (Fig. 3C).

The Y125 and S129 residues of α-syn are known as target amino acids for posttranslational modification, such as phosphorylation and nitration, and are also known as triggers of the formation of soluble toxic oligomers of α-syn. In our cell system, the Y125D and S129A mutants of α-syn did not exhibit cytotoxicity in the catecholaminergic PC12 cells (Fig. 3D,E), suggesting that the Y125 and S129 residues may play important roles in DA-related toxicity of α-syn.

Because methionine oxidation by DA is a possible target of DA-associated modification of α-syn, we investigated the effects of mutating the M116 and M127 residues of α-syn. DA-related toxicity decreased in the M116A mutant cells (Fig. 4C), and was nonexistent in the M127A and M116A/M127A mutant cells (Fig. 4D, E), therefore M127 may play a greater role in the DA-related toxicity of α-syn.

We also examined the oxidation of methionine residues in α-syn. As shown in Figure 5, Met(O) immunolabeling of α-syn was detected in wildtype PC12-TetOFF-α-syn cells with catecholamine metabolism, but much less so in cells treated with aMT, while treatment with reserpine, a vesicular monoamine transporter inhibitor that increases intracellular free DA, increased Met(O) labeling of α-syn (Fig. 5A). These results suggest that methionine oxidation of α-syn is affected by DA metabolism. Met(O) labeling was also much decreased in M127A and M116A/M127A mutant forms of α-syn, suggesting that M127 is the major target for oxidation by DA (Fig. 5B). Interestingly, α-syn prepared from Y125D and S129A mutant cells also showed decreased Met(O) compared with wildtype α-syn (Fig. 5C), suggesting that Y125 and S129, both of which contain a hydroxyl (-OH) group, may indirectly affect methionine oxidation. As a result, Y125D and S129A mutant cells may be resistant against DA-related toxicity.

Methionine residues are usually oxidized by H_2_O_2_ and then converted to Met(O). Therefore, we examined the production of H_2_O_2_ under several conditions. H_2_O_2_ was produced from DA *in vitro,* especially at alkaline pH. Moreover, co-incubation with the _125_YEMPS_129_ peptide enhanced the DA-related production of H_2_O_2_, while co-incubation with Y125- or S129-lacking peptides decreased the DA-related production of H_2_O_2_. We assume sulfide in the M127 may scavenge H_2_O_2_, thereby reducing free H_2_O_2_ levels. Because the M127A mutated YEAPS peptide has two hydroxyl structures on Y125 and S129 and therefore no H_2_O_2_ scavenging effects by M127, total H_2_O_2_ levels under the DA/YEAPS condition were higher than under the DA/YEMPS condition.

In conclusion, we summarize our results as follows: (i) The oligomerization of α-syn is enhanced in the presence of DA; (ii) DA or its metabolites may bind to sequences in the C-terminal region of α-syn, including the _125_YEMPS_129_ sequence; (iii) oxidative modification of methionine residues, especially M127, may be important for the DA-related toxicity of α-syn; (iv) DA can produce H_2_O_2_ especially under alkaline conditions and/or in the presence of _125_YEMPS_129_; and (v) the Y125 and S129 residues of α-syn, possibly via their hydroxyl structures, enhance DA-related oxidative modification of M127. Together, these results suggest a novel mechanism for the DA neuron-specific toxicity of α-syn, and the pathogenesis of PD, including in patients with the PARK4 family of mutations.
